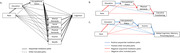# Occupational Status Versus Job Demands as Contributors to Racial Disparities in Cognitive Health

**DOI:** 10.1002/alz.089019

**Published:** 2025-01-09

**Authors:** Monica E Nelson, Brooke A. Huizenga, Vivian Ku, Mary Lesniak, Emily P. Morris, Jordan D Palms, Kiana A. Scambray, Ketlyne Sol, Lauren Taylor, Laura B. Zahodne

**Affiliations:** ^1^ University of Michigan, Ann Arbor, MI USA

## Abstract

**Background:**

Racial disparities in cognition persist even when accounting for traditional social factors. Occupational characteristics represent a less commonly measured socioeconomic factor that may contribute to health disparities through persistent workforce inequities. Socioeconomic status and cognitive stimulation are potential mechanisms that may link occupational characteristics to racial disparities in cognition. Thus, we aimed to assess whether occupational status (an indicator of socioeconomic status) or occupational complexity (an indicator of cognitive stimulation) mediated racial disparities in cognition.

**Methods:**

Participants were 493 Black or White older adults from the Michigan Cognitive Aging Project. Outcomes included episodic memory, language, processing speed, executive functioning, visuospatial functioning, and global cognition. Occupational characteristics included occupational status (U.S. Census categories) and occupational complexity (mental, social, and physical demands from O*NET). Income and wealth were considered as downstream mediators. Covariates included childhood health, parental education, and participant’s age, gender, and education. Mediation analyses assessed whether occupational status or occupational complexity explained racial disparities in cognition. Sequential mediation analyses assessed whether occupational status explained racial disparities in cognition through (a) occupational complexity, (b) income, and/or (c) wealth.

**Results:**

Occupational status partially mediated 5% of the association between Black race and memory (b = ‐.02, SE = .01, p = .045). Specifically, Black participants had lower occupational status than White participants, and lower occupational status related to worse memory. Occupational complexity did not mediate racial disparities in cognition. In sequential mediation analyses, racial disparities in executive functioning operated through inequities in education, occupational status, and physical job demands. Racial disparities in global cognition, memory, and processing speed operated through inequities in education, occupational status, and income.

**Conclusion:**

Occupation may influence cognitive disparities primarily through socioeconomic, rather than cognitive stimulation, pathways. Our study indicates the importance of considering upstream and downstream socioeconomic factors shaped by structural racism when assessing how occupational characteristics relate to cognition. Specifically, inequities in education seem to play a critical role in initiating occupation‐related cognitive disparities, which may operate through economic resources and job demands. Future work should explore other potential mechanisms underlying associations between occupational characteristics and cognition, assess longitudinal associations, and identify ways to address cognitive disparities related to workforce inequities.